# A Triple Mutation of BetaB2-Crystallin is Necessary to Develop Cataract and Glaucoma

**DOI:** 10.4172/2155-9570.1000690

**Published:** 2017-10-27

**Authors:** Anne Rübsam, Jennifer E Dulle, Sarah J Garnai, Hermant S Pawar, Patrice E Fort

**Affiliations:** 1 Department of Ophthalmology and Visual Sciences, University of Michigan, Ann Arbor, MI, USA; 2 Department of Molecular and Integrative Physiology, University of Michigan, Ann Arbor, MI, USA

**Keywords:** BetaB2-crystallin, Mutations, Lens epithelial cells, Retinal neurons

## Abstract

Crystallins are the predominant structural proteins in the lens that are evolutionarily related to stress proteins. There are two main crystallin gene families: α-crystallins and β/γ-crystallins. α- and β-crystallins were first considered to be lens-specific, but were recently recognized also as neuronal and retinal proteins. While in the ocular lens they are responsible for the maintenance of the transparency, their function in neurons is obviously different - regulating various protective mechanisms in degenerative conditions of the central nervous system. We recently reported the correlation between a gene conversion leading to a triple mutation in the betaB2-crystallin protein and a phenotype of familial congenital cataract with a high familial incidence also of primary open angle glaucoma. Congenital cataract is the leading cause of childhood blindness and progressive neuro degeneration of the optic nerve in glaucoma accounts as the leading cause of blindness worldwide. Altered solubility and stability of crystallin proteins cause cataract formation and are directly linked to a decrease in their protective function. Thus in this study, we evaluated the functional consequences of the mutations associated with this gene conversion on beta B2-crystallin protein biochemical properties in retinal neurons. We found that only the occurrence of the triple mutation leads to decreased solubility and formation of aggregates, which as we previously demonstrated, is associated with mislocalization to the mitochondria along with decreased mitochondrial function in retinal neurons and lens epithelial cells. Our data strongly support a significant role for beta B2-crystallin in both lenticular and retinal ocular tissues and warrant further analysis of its regulation and its impact not only in cataract formation but also in retinal neurodegenerative diseases.

## Introduction

We recently identified three simultaneous polymorphisms in the betaB2 (βB2)-crystallin protein in a family that demonstrated a genetically inherited form of early-onset cataracts [1]. Because over half of these family members also presented with glaucoma, we became interested in the functional consequences of these polymorphisms in lens epithelial cells and retinal neurons, both of which cell types that are responsible for the aforementioned disease conditions. While our initial analysis in both cell types focused only on the functional consequence of the triple mutant βB2-crystallin protein [2], this subsequent analysis aimed at characterizing the relative contribution of each amino acid conversion to the functional consequence previously reported: protein solubility and aggregate formation.

Crystallin proteins, including α-, β- and γ-crystallins, are the major water soluble proteins of the lens [3]. α-Crystallins are small heat shock proteins with chaperone function to inhibit protein aggregation. β/γ-Crystallins have been considered structural proteins with similar tertiary structures composing four Greek-key motifs divided into two domains. The major difference between β- and γ-crystallins is their oligomeric state: β-crystallins exist as homo- or hetero-oligomers, while γ-crystallins are exclusively monomers [4]. The integrity of the conserved Greek-key motifs plays a vital role in the structure, stability and function of β-crystallins. The human genome has an additional highly homologous βb2-crystallin pseudogene closely linked to the functional crystallin-βB2 locus, and transfer of information from the pseudogene to the active gene (gene conversion) results in congenital cataracts [1]. So far, fourteen mutations have been reported in βB2- crystallin, all of them in families with autosomal dominant cataract formation [5–13]. Previous studies have demonstrated that most mutations affecting crystallins would cause protein structure abnormality, resulting in an unstable protein that precipitates from solution and serves as a starting point for additional protein denaturation and precipitation, eventually resulting in cataract formation [14]. Indeed, crystallin mutations associated with cataract often lead to protein aggregation; however, little data has been reported for βB2-crystallin aggregation [9,15–17].

β/γ-Crystallins were recently found to be also expressed by hippocampal and retinal neurons and astrocytes [18]. While in lenticular tissue they are synergistically responsible for the maintenance of the lens transparency, their function in neurons is different. βB2-crystallin participates in the regulation of regenerative and degenerative conditions of the central nervous system (CNS), indicating its involvement in developmental injury and repair [19]. Accumulation of protein aggregates has been shown to be a primary cause also for various neurodegenerative diseases including glaucoma [20], while several studies have suggested a neuroprotective potential of βB2-crystallin when it comes to neuronal impairment *in vivo* and *in vitro* [18,21,22]. Thus, βB2-crystallin proteins might function as critical modulators in the course of neurodegenerative diseases like glaucoma and might be integral to glaucomatous neurodegeneration [22–24].

We previously demonstrated reduced solubility, increased aggregate formation, translocation to the mitochondria and reduced mitochondrial function of the βB2-crystallin triple mutant protein, that carries the three recently identified polymorphic gene conversions (R145W, Q147R and T150M), in retinal neurons and lens epithelial cells [2]. The purpose of this study was to analyze the effect of each of the three polymorphic mutations of βB2-crystallin alone or in combination on βB2-crystallin biochemical properties at first in retinal neurons.

## Methods and Materials

The βB2-crystallin cDNA was generated by RT-PCR using the High Capacity cDNA Reverse Transcription Kit (Applied Biosystems) using RNA from human retina and amplified using CRYBB2-specific primers. The PCR product was cloned into pGEM-T Easy, and then subcloned into pcDNA3.1. Three mutations, c.433 C>T (p.R145W), c. 440A>G (p.Q147R), and c.449C>T (p.T150M), were introduced into the wild type CRYBB2-carrying clone using the QuikChange Lightning kit (Agilent). Constructs were confirmed by sequencing.

R28 retinal neuronal cells were grown in DMEM containing 5 mM glucose supplemented with 10% fetal bovine serum (FBS) and differentiated to neurons on laminin-coated plates or coverslips with addition of cell-permeable cAMP (Sigma) as described previously [25]. The cells were plated on 6 well plates for the solubility analysis or on coverslips in 24 well plates for analysis by immunofluorescence (IF). 24h after seeding, cells were transfected using Lipofectamine 3000 (Thermo Fisher). Cells were harvested 48 h after transfection for the assays.

To analyze βB2-crystallin solubility, cells were lysed in Triton X-100 buffer containing 100 mM TRIS pH 7.5, 3 mM EGTA, 5 mM MgCl2, 0.5% Triton X-100, 1 mM PMSF with protease inhibitor cocktail (Roche). Cell lysates were sonicated, spun down for 3 min at 3000 g and the supernatant containing the soluble fraction was removed. The pellet containing the Triton X-100 insoluble protein was resuspended in 40μl Urea buffer (10mM Tris, pH 7.5, 0.1% Triton X-100, 1mM DTT, 5 mM MGCl2, 5 mM EGTA, 150 mM NaCL, 0.2mM PMSF and 9M urea). Protein assay was performed on the Triton X-100 soluble fractions using the Bradford method. Equal amounts of protein (30μg for the soluble fraction) were prepared and boiled under denaturing conditions for 10min at 70°C. Afterwards soluble fractions and equal volumes (40 μl) of the Triton X-100 non soluble fractions were analyzed by western blotting (WB). After separation by SDS-PAGE on a 4–12% gradient gel and 1h of transfer nitrocellulose membranes were incubated in a blocking buffer containing 4% milk/TBST for 1h and incubated with a goat primary antibody against βB2-crystallin (Santa Cruz) and a mouse primary antibody against β-Actin (Millipore) overnight at 4°C. Primary antibodies were detected using HRP conjugated secondary antibodies. Images were obtained using a chemoluminiscence detection device and analyzed using ImageJ software.

For immunofluorescent staining cells on coverslips were fixed in methanol/acetone (1:1) at −20°C to analyze the subcellular localization of βB2-crystallin. After permeabilization in 0.1% Triton-X solution and saturation in a blocking buffer containing 10% normal donkey serum and 1% BSA, all at room temperature, cells were incubated with a goat primary antibody against βB2-crystallin (Santa Cruz) overnight at 4°C. Primary antibody was detected using a secondary rabbit anti-goat IgG antibody coupled to Alexa fluorophore 488 (Jackson Immunoresearch). Finally, nuclei were counterstained using Hoechst nuclear dye (Life Technologies) before mounting. High resolution (1024x1024 pixel, 12 bit, 600Hz) Images were obtained using a Leica SP5 confocal microscope. Images were acquired using for betaB2- crystallin the Argon laser at 24% power, 458 nm excitation wavelength and 500 – 535 nm emission wavelength, 600–700V laser intensity and for Hoechst the 405 Laser Diode (50 mW), 405 nm excitation wavelength and 420 – 460 nm emission wavelength with 600 V laser intensity.

Each endpoint was assessed in 3 technical replicates and repeated in at least 2 independent experiments and p-values were assessed with GraphPAD Prism program using one-way ANOVA with Tukey’s post hoc analysis to correct for multiple comparisons.

## Results

**Figure 1:**
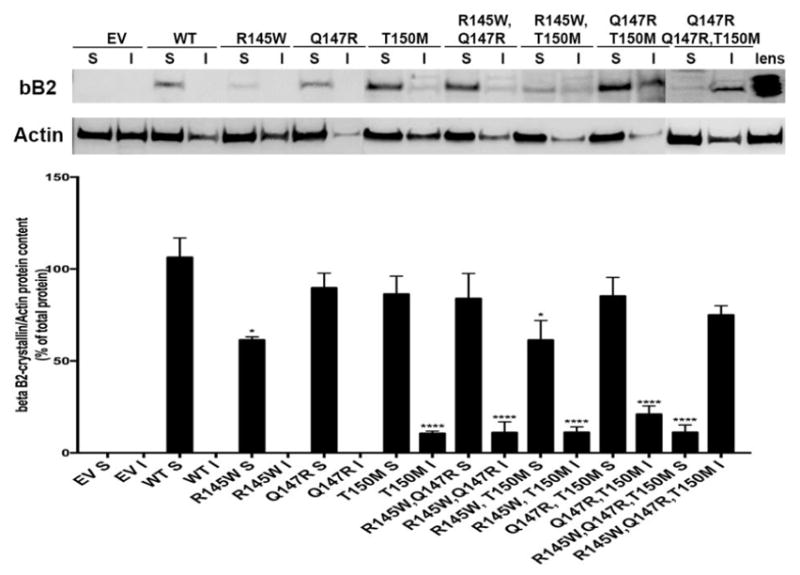
Only the triple mutation of betaB2 (βB2)-crystallin, associated with early-onset cataract and glaucoma, alter the protein solubility in retinal neurons. Representative Western Blot images and corresponding quantification of the solubility of wild-type (WT), single, double or triple mutation carrying βB2-crystallin proteins in retinal neurons (n ≥ 2). The specificity and sensitivity of the antibody against βB2-crystallin was ensured, as it resembles the signal obtained in an ocular lens sample. ^*^p<0.05 or ***p<0.0001 statistically significantly different from soluble WT using one-way ANOVA.

We examined the functional consequence of the three different βB2- crystallin mutations (R145W, Q147R and T150M) either by themselves or in double or triple combination, and compared them to the wild type (WT) βB2-crystallin. Protein solubility was assessed by a sequential Triton-based solubilization method. We found that the single and double βB2 mutants showed the same solubility profile as the WT βB2-crystallin, all of them remaining primarily in the soluble fractions in retinal neurons. Only the full gene conversion lead to a loss of solubility, shown by the triple mutant being found almost exclusively in the insoluble fraction (Figure 1). Interestingly, this effect is partially cell-type specific as we previously reported that when we expressed the triple mutant βB2-crystallin in lens epithelial cells, we observed a less dramatic shift in solubility of the triple mutant, with 35% of the protein remaining soluble [2].

**Figure 2:**
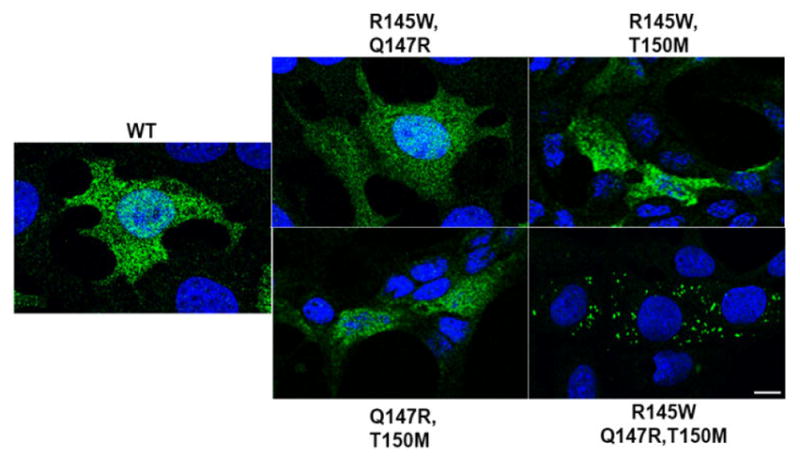
Only the triple mutation of betaB2 (βB2)-crystallin, associated with early-onset cataract and glaucoma, alter the aggregate formation in retinal neurons. Representative images of the cellular expression and aggregation patterns of the wild-type (WT) βB2-crystallin protein or the mutant βB2-crystallin proteins, containing either the single, double or triple mutation in retinal neurons (n ≥ 2). After transfection cells were analyzed by immunofluorescence (IF). Representative images of the IF signal obtained for βB2-crystallin (green) and Hoechst (nucleus, blue) are shown. (scale bar=10 μm).

Since we previously demonstrated that the full gene conversion triggered aggregate formation in retinal neurons, we decided to assess, how the individual mutations associated with this gene conversion were responsible for this phenomenon by performing immunohistological analysis. Immunofluorescent stainings were performed using an antibody against βB2-crystallin on cells overexpressing WT or one of the βB2-crystallin mutants. While the WT βB2-crystallin displayed a diffuse pattern of fluorescence in retinal neurons, the βB2-crystallin triple mutant exhibited a punctate pattern of fluorescence, often characteristic of decreased solubility (Figure 2). Consistent with the solubility data presented in Figure 1, the single and double βB2-crystallin mutants displayed a diffuse pattern indistinguishable from the one observed with the WT protein. The same staining patterns were observed for the βB2-crystallin WT and the triple βB2-crystallin mutant also in lens cells [2].

## Discussion

In the initial study on the identification of the three gene polymorphisms, 16 individuals with congenital cataracts from three generations of a family of Ashkenazi Jewish ancestry were recruited. Participants underwent ocular examinations and genomic DNA was extracted from peripheral blood. For further information on the genetic and sequencing analysis please review [1]. In a subsequent analysis we reported altered solubility, increased aggregation formation, mitochondrial translocation and consecutively mitochondrial dysfunction of this gene conversion leading to a triple mutation R145W, Q147R and T150M in the βB2-crystallin protein, that causes a familial congenital cataract phenotype and a high familial incidence of glaucoma [1,2]. Herein we wanted to elucidate the responsibility of each mutation in the overall phenotype. Specifically, we began assessing their respective impact on βB2-crystallin solubility and aggregate formation. Interestingly, the cumulative effect of the three mutations was necessary to observe reduced solubility and the formation of aggregates.

Mutations in crystallin proteins and their impact on the biochemical properties of these proteins has been extensively studied in the lens, where solubility and stability of the protein play critical roles in maintaining the optical transparency and altered structure or solubility results in cataract formation. It is known, that β/γ crystallins have a highly stable structure, consisting of four Greek key motifs folded into a β-sandwich structure [4]. Mutations associated with congenital cataract have been found in most regions of the βB2-crystallin protein, though ten of the fourteen reported mutations are located in Greek key motifs 3 and 4 [2]. Proteins can aggregate for a number of reasons including due to mutations affecting their structure. This is a very complex process, and various mutations may affect the overall structure through different mechanisms among which the following have been proposed: (i) directly decrease in protein solubility by the substitution of a charged or polar residue at the surface by a hydrophobic one; (ii) destabilization of the protein and leading to aggregation only when subjected to stresses and (iii) facilitation of aggregation by crosslinking of the molecules via intermolecular disulfide bonds [16,26,27].

Extensive studies during the last decade revealed changes in biochemical properties for many of the known mutations in the βB2- crystallin protein [9,10,28,29]. In most cases, single mutations on a single gene have been identified as causing cataract formation, however a few instances have been reported in which mutations on multiple genes were responsible [30]. Interestingly we observed, that only the introduction of the triple mutation leads to decreased solubility and aggregate formation. The behavior of the single or double mutants was equivalent to that of the WT protein. The identified three changes alter residues in the βB2-crystallin protein that are highly conserved across species, which reveals an important role of this region [1]. Computational predictions of the effects of each of the changes indicated that the gene conversions R145W and T150M are increasing hydrophobicity, while Q147R was predicted by PROVEAN and Polyphen-2 software to be neutral (Table 1) [1].

All three mutations are located at the last beta-strand of Greek-key motif 3 in βB2-crystalllin, suggesting that these three residues might not be directly involved in the core structure formation of βB2- crystalllin aggregates/fibrils. The data suggest that the full gene conversion and the triple mutation event associated is necessary to fully destabilize βB2-crystalllin, possibly by impairing the intermolecular binding ability. This would consequently disrupt the dimerization of the βB2-crystallin protein and impair the binding with other soluble proteins.

Additionally, accumulation of misfolded proteins can cause protein aggregation, especially in the aged brain, and these aggregates facilitate the formation of pathological amyloid deposits, a key event in several neurodegenerative disorders. Increased accumulation of aggregated proteins such as myocilin has been associated with glaucoma [31]. Moreover, it has been postulated, that unbound α-crystallin proteins, which are present in aqueous humor, contribute to the development of open angle glaucoma [32].

We recently demonstrated, that the triple gene conversion of βB2- crystallin not only leads to aggregate formation, but also to large insoluble aggregates that accumulate in the mitochondria, resulting in a loss of function [2]. Previous reports have shown that the βB2- crystallin protein is oxidized and localized to the mitochondria in photoreceptors isolated from experimental autoimmune uveitis (EAU)-induced mice [33,34]. βB2-crystallin has been shown to have chaperone functions distinct from its structural roles [35]. Thus aberrant localization of βB2-crystallin to the mitochondria may cause mitochondrial damage, loss of function, and subsequent induction of apoptosis. To date, the most significant non-structural role for βB2- crystallin elucidated has been in promoting axonal regeneration in the retina [36]. It has been long established that lens injury mitigates retinal ganglion cell loss and promotes retinal ganglion cell axon regeneration. Several studies reported that βB2-crystallin played a central role in this phenomenon. βB2-crystallin was found in the media of *ex vivo* retinas during axonal regeneration, and demonstrated to promote elongation *in vitro* when added to culture medium [22]. Furthermore, intravitreal injections of purified β-crystallins in retinal ganglion cells after axotomy or optic nerve crush in rats, resulted in increased survival of retinal ganglion cells [21]. It would be very revealing to assess how the gene conversion and the individual mutations associated would affect the neuroprotective effect of βB2- crystallin in these models.

Altogether, despite being obtained in cell lines with their inherent limitations, these results strongly suggest that the region of βB2- crystallin in which these mutations are found is critical for its role in lens epithelial cells as well as retinal neurons. Further in-depth investigations in the detailed consequences of these mutations are necessary to better understand the role of βB2-crystallin in lenticular and retinal cell survival.

## Figures and Tables

**Table 1:** Protein sequence and computational predictions of the three gene conversions on the betaB2-crystallin protein (adapted from 1).

Allele	C/T	A/G	C/T
Allele change	CGG ? TGG	CAG ? CGG	ACG ? ATG
Residue change	R (Arg) ? W (Trp)	Q (Gln) ? R (Arg)	T (Thr) ? M (Met)
Protein position	145	147	150
PROVEAN prediction	deleterious	neutral	deleterious
PolyPhen-2 interpretation	probably damaging	benign	probably damaging
